# Formal organocatalytic fluoronitromethane addition to tetrahydroisoquinolines through a CDC process

**DOI:** 10.3389/fchem.2026.1758992

**Published:** 2026-04-02

**Authors:** Ramon Rios, Luke Shirley, Marta Meazza

**Affiliations:** 1 Faculty of Chemistry, University of Southampton, Southampton, United Kingdom; 2 Department of Chemistry, Khalifa University, Abu Dhabi, United Arab Emirates

**Keywords:** cdc, desulfonylation, fluorine, Rose Bengal, tetrahydroquinolines

## Abstract

Recently, green chemistry has attracted significant attention due to societal challenges regarding waste generation and energy consumption. Consequently, cross-dehydrogenative couplings (CDC) have emerged as a premier strategy for C–C bond formation. In this work, we report a novel organocatalytic cascade methodology based on the CDC activation of tetrahydroisoquinolines, followed by the formal addition of fluoronitromethane. The reaction is catalyzed by organic dyes (Rose Bengal) using green LEDs as a light source and molecular oxygen as the stoichiometric oxidant, enabling the synthesis of fluorinated compounds in good yields.

## 1 Introduction

Sustainability has become one of the most critical challenges for organic chemists (Brahmachari, 2024). With raising pollution and the depletion of natural resources, the use of renewable energy and the minimization of waste are primary goals in method development. Photochemistry using organic dyes, a greener alternative to the current organometallic iridium and ruthenium photocatalysts (Martin et al., 2025; Patel and Singh, 2024), have emerged as a powerful tool to address these issues, utilizing light as a renewable energy source to drive complex transformations ranging from alkylations to cycloadditions (Amos et al., 2020; Bellotti et al., 2023; Ouies et al., 2024). On the other hand, the chemical community has long been interested in synthesizing fluorinated molecules (Ritter, 2018; Kalita et al., 2025; Yang et al., 2013). The strategic introduction of a fluorine atom into a target compound is paramount, as it allows for the modulation of physical properties and the enhancement of both metabolic and pharmacokinetic profiles (Gillis et al., 2015). The small size and high electronegativity of fluorine enable it to induce desirable effects, including conformational stabilization, modification of acid/base characteristics, and enhanced binding interactions (O’Hagan, 2010). Because these property enhancements are often interconnected, fluorinated compounds possess major commercial significance across several industries, including pharmaceuticals, agrochemicals, materials, and polymers (Hossain et al., 2018). Currently, approximately 18% of marketed pharmaceutical compounds contain a fluorine substituent, a figure that is exceeded in the agrochemical field (Meanwell, 2018).

**SCHEME 1:**
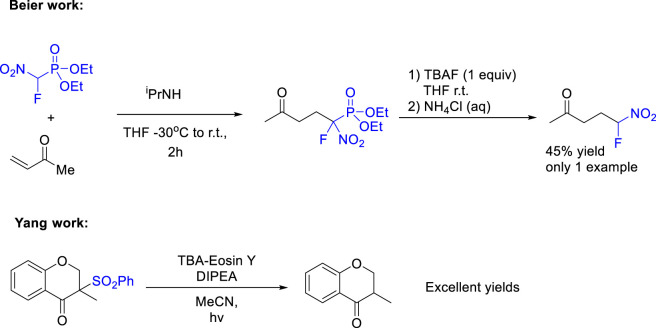
Previous Works.

In our research group, interested in Green Chemistry and Fluorine (Valero et al., 2011) we developed several organophotocatalytic reactions over last years. Taking advantage of the versatility and easy activation by light of organic dyes, we envisioned that organic dyes should be a greener alternative to the actual organometallic Iridium and Ruthenium photocatalysts. Based on the pioneering works of Rueping with Organic dyes (Rueping et al., 2013), we developed a fluoromalonate addition to tetrahydroisoquinolines and phosphoramidation both through a Cross Dehydrogenative Coupling (CDC) process (Shirley et al., 2016). For the same reasons stated before CDC have become a hot topic due to the lack of requirement for prefunctionalized starting materials and typically highly efficient atom economic processes, which should be an excellent way to shorten the common synthetic routes or introduce late-stage functionalization for interesting scaffolds. On the other hand, several methods have been developed for the introduction of fluorine atoms. Due to the difficulties to use molecular fluorine, the common strategies have been divided by direct substitution of an hydroxyl moiety by fluorine using DAST, the use of electrophilic fluorine (such as Select Fluor, NFSI, etc.). transition metal complexes with hypervalent fluorine reagents (Togni’s, Ruppert-Prakash, etc.) and nucleophilic building blocks that contain fluorine such as fluoromalonates, fluoromethylene (CFH_2_) fluoromethanebisulfones (CFSO_2_Ph)_2_, fluoroacetones, etc .,… However, despite the interest that could have the addition of simple fluoronitromethane (FNM) the direct addition of FNM to electrophiles has not been reported. Several groups (including ours) have reported the addition of α-fluoro-α-nitro (phenylsulfonyl) methane (FNSM); however, the desulfonylation of the final compound have never reported and by our experience is not so easy by the common available methods (Mg in MeOH, etc…). Only Beier and coworkers reported the formal addition of fluoronitromethane by using Diethyl fluoronitromethylphosphonate and treating the final compound with 1 equiv. of TBAF (one single example with moderate yields) for the dephosphorilation (Opekar et al., 2014). On the other hand, in 2013, Yang and coworkers reported a desulfonylation of 1,3 ketosulfones using TBA-Eosin Y as a catalyst and DIPEA as reducing agent with excellent results (Scheme 1).

**SCHEME 2:**
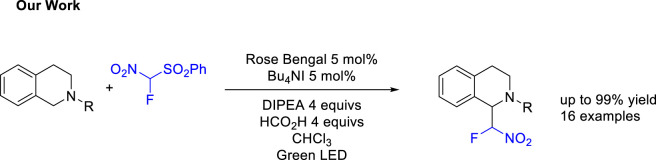
Our Work.

Inspired by those previous works, we envisioned a cascade reaction consisting of the addition of fluoronitro (phenylsulfonyl)methane to tetrahydroisoquinolines via a CDC process, coupled with a light-catalyzed desulfonylation to achieve the formal fluoronitromethane additions (Scheme 2).

## 2 Materials and methods

### 2.1 General procedure

To a solution of *N-*aryl-1,2,3,4-tetrahydroisoquinoline (0.05 mmol), 1-fluoro-1-nitro (phenylsulfonyl)methane (0.1 mmol), Rose Bengal (0.00025 mmol), and tetrabutylammonium iodide (0.00025 mmol) in chloroform (0.5 mL) were added diisopropylethylamine (0.2 mmol) and formic acid (0.2 mmol) in a screw-cap vial. The mixture was irradiated with green LEDs for 48h. The solvent was removed under reduced pressure, and the residue was purified by column chromatography using mixtures of increasing polarity of hexane/ethyl acetate to afford the desired product.

The X-Ray crystallographic coordinates for structures **4c**, **4f** and **13f** reported in this article have been deposited at the Cambridge Crystallographic Data Centre (CCDC) under deposition number CCDC 1819363 (for **13f**), 1819417 (for **4f**), and 1819425 (for **4c**). The data can be obtained free of charge from the Cambridge Crystallographic Data Centre via http://www.ccdc.cam.ac.uk/data_request/cif.

### 2.2 1-(fluoro (nitro)methyl)-2-phenyl-1,2,3,4-tetrahydroisoquinoline 4a. (13mg, yellow oil)

**Major Diastereoisomer:** ^**1**^ **H NMR (400 MHz, CDCl**_**3**_ **)** δ 7.39–7.29 (m, 3H, Ar), 7.27–7.17 (m, 2H, Ar), 7.05 (d, *J* = 8.2 Hz, 2H, Ar), 6.99 (d, *J* = 7.7 Hz, 1H, Ar), 6.92 (t, *J* = 7.3 Hz, 1H, Ar), 6.11 (dd, *J* = 50.6, 3.0 Hz, 1H, CHF), 5.50 (dd, *J* = 23.8, 3.0 Hz, 1H, CHN), 3.77 (dt, *J* = 11.3, 5.6 Hz, 1H, CH), 3.45 (ddd, *J* = 12.1, 8.8, 5.5 Hz, 1H, CH), 3.12 (ddd, *J* = 13.8, 8.3, 5.4 Hz, 1H, CH), 3.02 (dtd, *J* = 11.2, 5.9, 1.7 Hz, 1H, CH). ^**13**^ **C{1H} NMR (100 MHz, CDCl**_**3**_ **)** δ 148 (s, Cq), 136.8 (s, Cq), 130.0 (s, CH), 129.2 (s, CH), 128.7 (s, Cq), 128.4 (d, *J* = 1.7 Hz, Cq), 127.9 (s, Cq), 126.9 (s, CH), 119.7 (s, CH), 114.0 (s, CH), 110.53 (d, J = 246.9 Hz, CHF), 61.8 (d, *J* = 20.5 Hz, CH_2_), 43.6 (s, CH_2_), 27.8 (d, *J* = 3.6 Hz, CH_2_). ^**19**^ **F NMR (376 MHz, CDCl**_**3**_ **)** δ −156.05 (dd, *J* = 50.6, 23.8 Hz). **HRMS *m/z* (ESI+):** Exact mass calculated for C_16_H_16_FN_2_O_2_ [M + H]^+^ 286.1117, found 286.1119. **IR (NaCl, liquid film)** 2,920, 2,359, 1,569, 1,146 cm^-1^.

**Minor Diastereoisomer**: ^**1**^ **H NMR (400 MHz, CDCl**_**3**_ **)** δ 7.25–7.12 (m, 6H, Ar), 6.87 (d, *J* = 8.2 Hz, 2H, Ar), 6.81 (t, *J* = 7.3 Hz, 1H, Ar), 6.04 (dd, *J* = 49.8, 3.8 Hz, 1H, CHF), 5.41 (dd, *J* = 22.3, 3.6 Hz, 1H, CHN), 3.76 (ddd, *J* = 13.8, 9.8, 4.4 Hz, 1H, CH), 3.59 (dt, *J* = 13.1, 5.0 Hz, 1H, CH), 2.96 (ddd, *J* = 15.6, 9.8, 5.5 Hz, 1H, CH), 2.72 (dt, *J* = 16.2, 4.4 Hz, 1H, CH). ^**13**^ **C{1H}NMR (100 MHz, CDCl**_**3**_ **)** δ 154.52 (s, Cq), 133.54 (d, *J* = 794.1 Hz, Cq), 129.52 (s, Cq), 129.41 (s, CH), 128.78 (s, CH), 128.58 (s, CH), 127.08 (s, CH), 126.92 (s, CH), 121.94 (s, CH), 120.39 (s, CH), 117.65 (s, CH), 116.41 (s, CH), 105.37 (d, *J* = 359.8 Hz, CHF), 61.12 (d, *J* = 19.0 Hz, CHN), 53.27 (s, CH_2_), 25.80 (s, CH_2_). ^**19**^ **F NMR (376 MHz, CDCl**_**3**_ **)** δ −152.10 (dd, *J* = 49.8, 22.1 Hz). **HRMS *m/z* (ESI+):** Exact mass calculated for C_16_H_16_FN_2_O_2_ [M + H]^+^ 286.1117, found 286.1119.

### 2.3 1-(fluoro (nitro)methyl)-2-(p-tolyl)-1,2,3,4-tetrahydroisoquinoline 4b (yellow foam, 14.1 mg)

**Major diastereoisomer**: ^**1**^ **H NMR (400 MHz, CDCl**_**3**_ **)** δ 7.31 (td, *J* = 7.5, 1.1 Hz, 1H, Ar), 7.24 (d, *J* = 7.2 Hz, 1H, Ar), 7.20 (t, *J* = 7.8 Hz, 1H, Ar), 7.15 (d, *J* = 8.3 Hz, 2H, Ar), 6.99 (d, *J* = 7.5 Hz, 1H, Ar), 6.95 (d, *J* = 8.6 Hz, 2H, Ar), 6.08 (dd, *J* = 50.7, 3.2 Hz, 1H, CHF), 5.43 (dd, *J* = 23.5, 3.1 Hz, 1H,CHN), 3.72 (dt, *J* = 11.3, 5.6 Hz, 1H, CH), 3.44 (dt, *J* = 11.3, 5.6 Hz, 1H, CH), 3.12–2.95 (m, 2H, CH_2_), 2.29 (s, 3H, CH_3_). ^**13**^ **C{1H} NMR (100 MHz, CDCl**_**3**_ **)** δ 145.7 (s, Cq), 136.6 (s, Cq), 130.3 (s), 129.2 (s), 128.9 (s), 128.6 (s), 128.3 (d, *J* = 1.7 Hz), 127.7 (s), 126.6 (s), 114.7 (s, CH), 110.6 (d, *J* = 246.8 Hz, CHF), 61.8 (d, *J* = 20.4 Hz, CHN), 43.6 (s, CH_2_), 27.6 (d, *J* = 3.1 Hz, CH_2_), 20.3 (s, CH_3_). ^**19**^ **F NMR (376 MHz, CDCl**_**3**_ **)** δ −155.39 (dd, *J* = 50.6, 23.4 Hz). **HRMS *m/z* (ESI+):** Exact mass calculated for C_17_H_18_FN_2_O_2_ [M + H]^+^ 301.1354, found 301.1347. **IR (NaCl, liquid film)** 2,924, 2,360, 1717, 1,215 cm.^-1^

**Minor diastereoisomer**. ^**1**^ **H NMR (400 MHz, CDCl**_**3**_ **)** δ 7.28 (dd, *J* = 6.8, 2.3 Hz, 1H, Ar), 7.25–7.13 (m, 3H, Ar), 7.05 (d, *J* = 8.2 Hz, 2H, Ar), 6.84 (d, *J* = 8.6 Hz, 2H, Ar), 6.10 (dd, *J* = 49.8, 3.5 Hz, 1H, CHF), 5.42 (dd, *J* = 23.3, 3.8 Hz, 1H, CHN), 3.80 (ddd, *J* = 13.9, 9.8, 4.3 Hz, 1H, CH), 3.60 (dt, *J* = 13.5, 5.0 Hz, 1H, CH), 2.99 (ddd, *J* = 15.4, 9.4, 5.1 Hz, 1H, CH), 2.74 (dt, *J* = 16.5, 4.2 Hz, 1H, CH), 2.25 (s, 3H, CH_3_). ^**13**^ **C{1H}NMR (100 MHz, CDCl**_**3**_ **)** δ 147.3 (s, Cq), 136.9 (s, Cq), 130.3 (s, CH), 130.1 (s, Cq), 129.7 (s, CH), 129.3 (s, CH), 128.6 (s, Cq), 127.0 (s, CH), 126.9 (s, CH), 117.4 (s, CH), 113.1 (d, *J* = 247.3 Hz, CHF), 61.4 (d, *J* = 18.9 Hz, CHN), 44.9 (d, *J* = 4.4 Hz, CH_2_), 25.7 (s,CH_2_), 20.5 (s, CH_3_). ^**19**^ **F NMR (376 MHz, CDCl**_**3**_ **)** δ −152.53 (dd, *J* = 49.8, 23.1 Hz). **HRMS *m/z* (ESI+):** Exact mass calculated for C_17_H_18_FN_2_O_2_ [M + H]^+^ 301.1354, found 301.1347.

### 2.4 1-(fluoro (nitro)methyl)-2-(4-fluorophenyl)-1,2,3,4-tetrahydroisoquinoline 4c. (White solid, mp = 85–95^o^C, 15.2 mg)

**Major diastereoisomer (white solid, mp = 85-95** **°C):** ^**1**^ **H NMR (400 MHz, CDCl**_**3**_ **)** δ 7.33 (td, *J* = 7.5, 1.3 Hz, 1H, Ar), 7.26–7.18 (m, 2H, Ar), 7.07–7.00 (m, 3H, Ar), 7.00–6.93 (m, 2H, Ar), 6.04 (dd, *J* = 50.6, 3.6 Hz, 1H, CHF), 5.35 (dd, *J* = 22.3, 3.5 Hz, 1H, CHN), 3.70 (dt, *J* = 12.1, 6.0 Hz, 1H, CH), 3.43 (dt, *J* = 12.0, 6.2 Hz, 1H, CH), 3.08–2.95 (m, 2H, CH_2_). ^**19**^ **F NMR (376 MHz, CDCl**_**3**_ **)** δ −154.55 (dd, *J* = 50.6, 22.4 Hz). ^**13**^ **C{1H} NMR (100 MHz, CDCl**_**3**_ **)** δ 157.22 (d, *J* = 239.8 Hz, CqF), 144.63 (d, *J* = 2.3 Hz, Cq), 136.30 (s, Cq), 129.04 (s, CH), 128.75 (s, CH), 128.19 (s, CH), 127.74 (d, *J* = 1.7 Hz, Cq), 126.76 (s), 116.47 (d, *J* = 7.6 Hz, CH), 116.22 (d, *J* = 22.3 Hz, CH), 110.66 (d, *J* = 246.6 Hz, CHF), 62.05 (d, *J* = 20.7 Hz, CHN), 44.26 (s, CH_2_), 27.23 (d, *J* = 2.7 Hz, CH_2_). **HRMS *m/z* (ESI+):** Exact mass calculated for C_16_H_15_F_2_N_2_O_2_ [M + H]^+^ 305.1101, found 305.1108. **IR (NaCl, liquid film)** 2,924, 2,359, 1,571, 1,508, 1,235 cm.^-1^

**Minor Diastereoisomer:** ^**1**^ **H NMR (400 MHz, CDCl**_**3**_ **)** δ 7.33–7.27 (m, 2H, Ar), 7.26–7.19 (m, 2H, Ar), 6.96–6.91 (m, 2H, Ar), 6.90–6.84 (m, 2H, Ar), 6.09 (dd, *J* = 49.7, 3.5 Hz, 1H, CHF), 5.35 (dd, *J* = 23.6, 3.3 Hz, 1H, CHN), 3.80 (ddd, *J* = 14.4, 10.4, 4.1 Hz, 1H, CH), 3.60 (dt, *J* = 13.4, 4.8 Hz, 1H, CH), 2.96 (ddd, *J* = 16.1, 10.1, 5.8 Hz, 1H, CH), 2.74 (dt, *J* = 16.3, 4.0 Hz, 1H, CH). ^**19**^ **F NMR** (376 MHz, CDCl_3_) δ −153.41 (dd, *J* = 49.7, 23.6 Hz).

### 2.5 2-(3-chlorophenyl)-1-(fluoro (nitro)methyl)-1,2,3,4-tetrahydroisoquinoline 4d. (Yellow foam, 10.2 mg)

**Major diastereoisomer**: ^**1**^ **H NMR (400 MHz, CDCl**_**3**_ **)** δ 7.34 (ddd, J = 10.6, 8.0, 1.4 Hz, 2H, Ar), 7.29–7.24 (m, 2H, Ar), 7.22 (t, *J* = 7.3 Hz, 1H, Ar), 7.02–6.98 (m, 2H, Ar), 6.93 (dd, *J* = 8.4, 2.6 Hz, 1H, Ar), 6.88 (dd, *J* = 7.9, 1.1 Hz, 1H, Ar), 6.09 (dd, *J* = 50.5, 3.0 Hz, 1H, CHF), 5.45 (dd, *J* = 23.5, 2.9 Hz, 1H, CHN), 3.75 (dt, *J* = 11.1, 5.5 Hz, 1H, CH), 3.42 (ddd, *J* = 10.9, 9.2, 5.1 Hz, 1H, CH), 3.14 (ddd, *J* = 14.7, 8.7, 5.6 Hz, 1H, CH), 3.02 (dtd, *J* = 16.0, 5.3, 1.7 Hz, 1H, CH). ^**13**^ **C{1H} NMR (100 MHz, CDCl**_**3**_ **)** δ 148.9 (s, Cq), 136.3 (s, Cq), 135.7 (s, Cq), 130.7 (s, CH), 129.3 (s, CH), 128.5 (s, CH), 128.0 (d, *J* = 1.7 Hz, Cq), 127.9 (s, CH), 127.8 (s, CH), 126.9 (s, CH), 119.3 (s, CH), 113.7 (s, CH), 111.5 (s, CH), 110.1 (d, *J* = 247.2 Hz, CHF), 61.5 (d, *J* = 20.8 Hz, CHN), 43.5 (s, CH_2_), 27.6 (d, *J* = 3.9 Hz, CH_2_). ^**19**^ **F NMR (376 MHz, CDCl**_**3**_ **)** δ −156.22 (dd, *J* = 50.5, 23.5 Hz). **HRMS *m/z* (ESI+):** Exact mass calculated for C_16_H_15_ClFN_2_O_2_ [M + H]^+^ 321.0798, found 321.0801. **IR (NaCl, liquid film)** 3,020, 2,929, 1,593, 1,213 cm.^-1^

**Minor diastereoisomer (yellowish foam)**: ^**1**^ **H NMR (400 MHz, CDCl**_**3**_ **)** δ 7.26–7.20 (m, 2H, Ar), 7.18–7.12 (m, 2H, Ar), 7.09 (t, *J* = 8.2 Hz, 1H, Ar), 6.84 (t, *J* = 2.2 Hz, 1H, Ar), 6.76 (td, *J* = 8.5, 2.3 Hz, 2H, Ar), 6.02 (dd, *J* = 49.8, 3.9 Hz, 1H, CHF), 5.38 (dd, *J* = 21.2, 4.1 Hz, 1H, CHN), 3.76 (ddd, *J* = 13.6, 9.4, 4.6 Hz, 1H, CH), 3.61–3.52 (m, 1H, CH), 3.00–2.90 (m, 1H, CH), 2.75 (dt, *J* = 16.4, 4.7 Hz, 1H, CH). ^**19**^ **F NMR (376 MHz, CDCl**_**3**_ **)** δ −151.88 (dd, *J* = 49.8, 21.4 Hz). **HRMS *m/z* (ESI+):** Exact mass calculated for C_16_H_15_ClFN_2_O_2_ [M + H]^+^ 321.0798, found 321.0801.

### 2.6 2-(4-chlorophenyl)-1-(fluoro (nitro)methyl)-1,2,3,4-tetrahydroisoquinoline 4e. (Yellow foam, 15.7 mg)

**Major diastereoisomer:** ^**1**^ **H NMR (400 MHz, CDCl**_**3**_ **)** δ 7.33 (td, *J* = 7.5, 1.3 Hz, 1H, Ar), 7.29 (d, *J* = 9.2 Hz, 2H, Ar), 7.27–7.19 (m, 2H, Ar), 7.01 (d, *J* = 7.6 Hz, 1H, Ar), 6.95 (d, *J* = 9.1 Hz, 2H, Ar), 6.07 (dd, *J* = 50.6, 3.2 Hz, 1H, CHF), 5.41 (dd, *J* = 23.1, 3.1 Hz, 1H, CHN), 3.73 (dt, *J* = 11.4, 5.7 Hz, 1H, CH), 3.42 (ddd, *J* = 11.8, 8.2, 5.3 Hz, 1H, CH), 3.10 (ddd, *J* = 13.9, 7.9, 5.6 Hz, 1H, CH), 3.01 (dtd, *J* = 16.3, 5.8, 1.6 Hz, 1H, CH). ^**13**^ **C{1H} NMR (100 MHz, CDCl**_**3**_ **)** δ 146.6 (s, Cq), 136.5 (s, Cq), 129.8 (s, CH), 129.3 (s, CH), 128.7 (s, CH), 128.1(d, *J* = 1.7 Hz, Cq), 127.9 (d, *J* = 1.0 Hz, CH), 127.0 (s, CH), 124.8 (s, Cq), 115.3 (s, CH), 110.4 (d, *J* = 247.0 Hz, CHF), 61.8 (d, *J* = 20.9 Hz, CHN), 43.8 (s, CH_2_), 27.7 (d, *J* = 3.5 Hz, CH_2_). ^**19**^ **F NMR (376 MHz, CDCl**_**3**_ **)** δ −155.65 (dd, *J* = 50.6, 23.1 Hz). **HRMS *m/z* (ESI+):** Exact mass calculated for C_16_H_15_ClFN_2_O_2_ [M + H]^+^ 321.0798, found 321.0802. **IR (NaCl, liquid film)** 2,925, 2,359, 1,595, 1,569, 1,255 cm.^-1^

**Minor diastereoisomer**: ^**1**^ **H NMR (400 MHz, CDCl**_**3**_ **)** δ 7.30 (m, 2H), 7.22 (m, 2H), 7.19 (d, *J* = 9.1 Hz, 2H), 6.86 (d, *J* = 9.0 Hz, 2H), 6.08 (dd, *J* = 49.8, 3.9 Hz, 1H), 5.41 (dd, *J* = 22.0, 4.3 Hz, 1H), 3.82 (ddd, *J* = 13.8, 9.7, 4.5 Hz, 1H), 3.60 (dt, *J* = 13.3, 5.1 Hz, 1H), 3.00 (ddd, *J* = 15.5, 9.6, 5.5 Hz, 1H), 2.79 (dt, *J* = 16.8, 4.7 Hz, 1H). ^**13**^ **C NMR (100 MHz, CDCl**_**3**_ **)** δ 148.0 (s, Cq), 136.5 (s, Cq), 129.7 (s, CH), 129.4 (s, CH), 129.0 (d, *J* = 1.5 Hz, Cq), 128.9 (s, CH), 127.1 (s, CH), 127.0 (s, CH), 125.6 (s), 117.9 (s, CH), 112.9 (d, *J* = 248.1 Hz, CHF), 61.2 (d, *J* = 18.9 Hz, CHN), 44.7 (d, *J* = 4.3 Hz, CH_2_), 25.8 (s, CH_2_). ^**19**^ **F NMR (376 MHz, CDCl**_**3**_ **)** δ −152.34 (dd, *J* = 49.8, 22.0 Hz). **HRMS *m/z* (ESI+):** Exact mass calculated for C_16_H_15_ClFN_2_O_2_ [M + H]^+^ 321.0798, found 321.0802.

### 2.7 1-(fluoro (nitro)methyl)-2-(4-(trifluoromethyl)phenyl)-1,2,3,4-tetrahydroisoquinoline 4f. (Yellow solid, mp:125–135^o^C, 17.2 mg)

**Major diastereoisomer:** ^**1**^ **H NMR (400 MHz, CDCl**_**3**_ **)** δ 7.50 (d, *J* = 8.7 Hz, 2H, Ar), 7.36–7.18 (m, 4H, Ar), 6.99 (d, *J* = 8.8 Hz, 2H, Ar), 6.10 (dd, *J* = 49.9, 4.3 Hz, 1H, CHF), 5.55 (dd, *J* = 20.1, 4.3 Hz, 1H, CHN), 3.88 (ddd, *J* = 13.4, 8.8, 4.8 Hz, 1H, CH), 3.63 (dt, *J* = 12.8, 5.6 Hz, 1H, CH), 3.00 (ddd, *J* = 14.4, 8.5, 5.3 Hz, 1H, CH), 2.90 (dt, *J* = 16.4, 5.1 Hz, 1H, CH). ^**13**^ **C{1H}NMR (100 MHz, CDCl**_**3**_ **)** δ 150.09 (s), 136.40 (s), 129.59 (s), 128.61 (s), 127.93 (s), 127.24 (d, *J* = 3.8 Hz), 127.16 (s), 124.74 (q, *J* = 270.6 Hz), 121.02 (q, *J* = 33.0 Hz), 112.53 (s), 110.01 (d, *J* = 247.4 Hz), 61.45 (d, *J* = 21.0 Hz), 43.61 (s), 27.88 (d, *J* = 4.4 Hz). ^**19**^ **F NMR (376 MHz, CDCl**_**3**_ **)** δ −61.78 (s, CF_3_), −151.37 (dd, *J* = 49.9, 20.1 Hz, CF). **HRMS *m/z* (ESI+):** Exact mass calculated for C_17_H_15_F_4_N_2_O_2_ [M + H]^+^ 355.1064, found 355.1065.

**Minor diastereoisomer** ^**1**^ **H NMR (400 MHz, CDCl**_**3**_ **)** δ 7.59 (d, *J* = 8.8 Hz, 2H, Ar), 7.35 (td, *J* = 7.5, 0.9 Hz, 1H, Ar), 7.30–7.20 (m, 3H, Ar), 7.08 (d, *J* = 8.8 Hz, 2H, Ar), 7.01 (d, *J* = 7.6 Hz, 1H, Ar), 6.11 (dd, *J* = 50.5, 2.9 Hz, 1H, CHF), 5.55 (dd, *J* = 23.6, 2.8 Hz, 1H, CHN), 3.83 (dt, *J* = 10.8, 5.2 Hz, 1H, CH), 3.47 (td, *J* = 10.5, 5.0 Hz, 1H, CH), 3.20 (ddd, *J* = 15.5, 9.5, 5.7 Hz, 1H, CH), 3.04 (dtd, *J* = 15.9, 4.9, 1.9 Hz, 1H, CH). ^**13**^ **C NMR (100 MHz, CDCl**_**3**_ **)** δ 150.1 (s, Cq), 136.4 (s, Cq), 129.6 (s, CH), 128.6 (s, CH), 127.9 (s, CH), 127.9 (m, CH), 127.2 (q, *J* = 3.9 Hz, CH), 127.2 (s, Cq), 124.7 (q, *J* = 270.6 Hz, CF_3_), 121.02 (q, *J* = 33.0 Hz, Cq) 112.5 (s, CH), 110.0 (d, *J* = 247.4 Hz, CHF), 61.5 (d, *J* = 21.0 Hz, CHN), 43.6 (s, CH_2_), 27.9 (d, *J* = 4.4 Hz, CH_2_). ^**19**^ **F NMR (376 MHz, CDCl**_**3**_ **)** δ −61.65 (s, CF_3_), −156.49 (dd, *J* = 50.5, 23.6 Hz, CF). **HRMS *m/z* (ESI+):** Exact mass calculated for C_17_H_15_F_4_N_2_O_2_ [M + H]^+^ 355.1064, found 355.1065. **IR (NaCl, liquid film)** 3,018, 2,359, 1,574, 1,214 cm.^-1^

### 2.8 1-(fluoro (nitro)methyl)-2-(4-methoxyphenyl)-1,2,3,4-tetrahydroisoquinoline 4g (yellow oil 14.9 mg)

**Major diastereoisomer**: ^**1**^ **H NMR (400 MHz, CDCl**_**3**_ **)** δ 7.31 (td, *J* = 7.6, 1.1 Hz, 1H, Ar), 7.25–7.17 (m, 2H, Ar), 7.01 (d, *J* = 8.3 Hz, 1H, Ar), 6.98 (d, *J* = 9.2 Hz, 2H, Ar), 6.89 (d, *J* = 9.2 Hz, 2H, Ar), 6.04 (dd, *J* = 50.7, 3.6 Hz, 1H, CHF), 5.32 (dd, *J* = 22.4, 3.6 Hz, 1H, CHN), 3.78 (s, 3H, OCH_3_), 3.72–3.63 (m, 1H, CH), 3.42 (dt, *J* = 12.5, 6.0 Hz, 1H, CH), 2.99 (t, *J* = 6.1 Hz, 1H, CH). ^**13**^ **C NMR (100 MHz, CDCl**_**3**_ **)** δ 154.3 (s, Cq), 142.5 (s, Cq), 136.6 (s, Cq), 129.0 (s, CH), 128.5 (s, Cq), 127.8 (d, *J* = 1.6 Hz, Cq), 126.8 (s, CH), 117.8 (s, CH), 115.2 (s, CH), 111.0 (d, *J* = 246.5 Hz, CHF), 62.4 (d, *J* = 20.5 Hz, CHN), 55.8 (s, OCH_3_), 44.6 (s, CH_2_), 27.4 (d, *J* = 2.1 Hz, CH_2_). ^**19**^ **F NMR (376 MHz, CDCl**_**3**_ **)** δ −154.14 (dd, *J* = 50.7, 22.4 Hz). **HRMS *m/z* (ESI+):** Exact mass calculated for C_17_H_18_FN_2_O_3_ [M + H]^+^ 317.1289, found 317.1296. **IR (CHCl**_**3**_ **, NaCl, liquid film)** 2,924, 2,361, 1718, 1,508, 1,214 cm.^-1^

**Minor diastereoisomer** ^**1**^ **H NMR (400 MHz, CDCl**_**3**_ **)** δ 7.33–7.27 (m, 2H, Ar), 7.25–7.19 (m, 2H, Ar), 6.90–6.84 (m, 2H, Ar), 6.81–6.76 (m, 2H, Ar), 6.08 (dd, *J* = 49.7, 3.3 Hz, 1H, CHF), 5.41–5.23 (m, 1H, CHN), 3.78–3.72 (m, 1H, CH), 3.75 (s, 3H, OCH_3_), 3.50 (ddd, *J* = 13.5, 4.9, 3.9 Hz, 1H, CH), 3.03–2.87 (m, 1H, CH), 2.69 (dt, *J* = 16.8, 4.0 Hz, 1H, CH). ^**19**^ **F NMR (376 MHz, CDCl**_**3**_ **)** δ −153.75 (dd, *J* = 49.7, 24.4 Hz). **HRMS *m/z* (ESI+):** Exact mass calculated for C_17_H_18_FN_2_O_3_ [M + H]^+^ 317.1289, found 317.1296.

## 3 Results and discussion

### 3.1 Reaction optimization

Following preliminary results, an extensive optimization study was conducted. We screened solvents, photocatalysts, amine additives, and phase transfer catalysts (Tables 1–3). As summarized in Table 1, chloroform proved superior to acetonitrile, providing full conversion. Regarding the photocatalyst, both Rose Bengal and Eosin Y delivered comparable diastereomeric ratios (d.r.) and conversions; however, Rose Bengal was selected for subsequent steps. Remarkably several LED wavelengths have been tested being Green wavelength which gave better results as previously reported in our group in similar reactions (Shirley et al., 2016).

**TABLE 1:** Screening of photocatalyst.

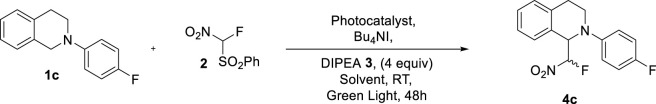					
Entry	Solvent	Photocatalyst	d.r^b^	Conversion (%)	Yield (%)^a^
1	CHCl_3_	Rose Bengal **I**	2:1	100	50
2	MeCN	Rose Bengal **I**	2:1	66	ND
3	CHCl_3_	Eosin Y **II**	2:1	90	48
4	CHCl_3_	4CZIPN^b^ **III**	ND	0	ND

Reaction conditions: TBAI (5 mol%), photocatalyst (5 mol%), amine (2 equiv.), rt, green LED.

^a^ Determined by crude NMR.

^b^ Must in d.r.

**TABLE 2:** Screening of amine additives.

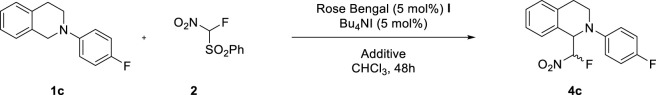			
Entry	Additive^a^	Conversion (%)^e^	Yield (%)^f^ ^,^ ^g^
1	NEt_3_ (**5**)	100	64
2	DIPEA (**3**)	100	94
3	DIPMA (**6**)	100	73
4	TMEDA (**7**)	100	35
5	TBA (**8**)	100	75
6	Hantzsch ester (**9**)	100	74
7	1,3 dinitrobenzene (**10**)	100	0
8	DIPEA (**3**)/formic acid (**11**)^b^ ^,^ ^c^	100	<99
9	DIPEA (**3**)/formic acid (**11**)^b^ ^,^ ^d^	100	<99
10	DBU (**12**)/FA (**11**)	NA	NA
11	TBA (**8**)/FA (**11**)	100	96

^a^ 4 equivalents of additive used.

^b^ 4 equivalents each of DIPEA, and formic acid were added.

^c^ additive added after FNSM, addition was completed.

^d^ DIPEA, and formic acid added with reagents.

^e^ Conversion monitored by TLC, and 1H NMR.

^f^ isolated yield after purification via column chromatography. DIPEA, Diisopropylethylamine; DIPMA, Diisoproplymethylamine; TMEDA, Tetramethylethylenediamine; TBA, Tributylamine, Hantzsch ester - Diethyl 1,4-dihydro-2, 6-dimethyl-3, 5-pyridine dicarboxylate.

^g^ Reactions done using 0.05 mmol 1c.

**TABLE 3:** Screening of phase transfer catalyst and its loading.

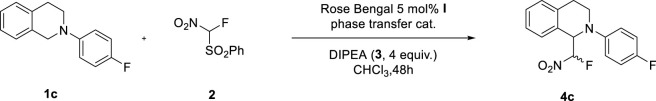			
Entry	Phase transfer Cat	Loading (mol%)	Conversion (%)
1	Bu_4_NOAc	20	35
2	Bu_4_NBr	20	<5
3	Bu_4_NI	20	100
4	Bu_4_NI	15	100
5	Bu_4_NI	10	100
6	Bu_4_NI	5	100
7	—	0	0
8	Bu_4_NI^b^	5	10

^a^ Crude NMR, was used to determine d.r and conversion. by comparing the benzylic CH, protons on the C1 carbon adjacent the nitrogen.

^b^ Reactions carried without Oxigen.

^c^ Reactions done using 0.05 mmol 1c.

Screening of Amine Additives: The role of the amine additive as a sacrificial hydrogen donor for the reduction of the sulfonyl group was investigated next. As detailed in Table 2, most amine additives facilitated the consumption of the tetrahydroisoquinoline starting materials. While Diisopropylethylamine (DIPEA, **3**), Diisopropylmethylamine (DIPMA, **6**), and Hantzsch ester (**9**) all provided excellent conversions, triethylamine (**5**) and TMEDA (**7**) resulted in significantly lower yields. Notably, the use of 1,3-dinitrobenzene (**10**) resulted in full conversion to the sulfonylated intermediate but inhibited formation of the final desulfonated product (**4**).Although the combination of DIPEA (**3**) or Tributylamine (TBA, **8**) with formic acid (**11**) accelerated the reaction time from 48 h to 20 h, DIPEA (**3**) was selected as the optimal additive. This choice was driven by its proven utility in photochemical transformations and superior safety profile compared to TBA.

The influence of the phase transfer catalyst and its loading was subsequently examined (Table 3). The counter-anion proved critical; tetrabutylammonium acetate resulted in poor conversion, while the bromide salt led to degradation of materials (<5% conversion). In contrast, tetrabutylammonium iodide (TBAI) provided full conversion. Control experiments confirmed that the reaction does not proceed in the absence of the catalyst (Entry 7). Further optimization of the loading revealed that 5 mol% of TBAI was sufficient to maintain quantitative conversion.

Finally, the effect of external additives was evaluated. The inclusion of 4Å molecular sieves or sparging the reaction mixture with an oxygen atmosphere provided no tangible improvement in yield compared to the standard conditions. Consequently, the optimized conditions were established as CHCl_3_ solvent, Rose Bengal photocatalyst, DIPEA additive, and 5 mol% TBAI under green LED irradiation.

### 3.2 Scope of the reaction

**SCHEME 3:**
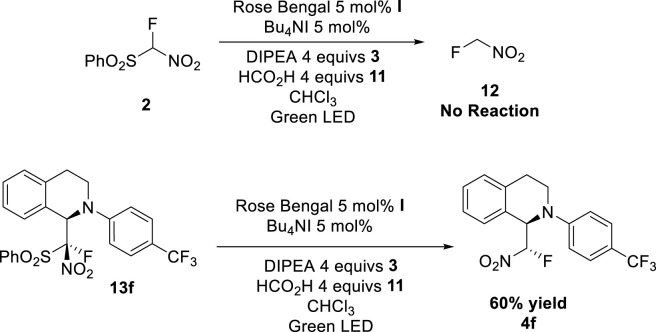
Mechanistic Studies.

After the optimization, we found that the best conditions were, the use of Rose Bengal as organic dye (5 mol%), Bu_4_NI as phase transfer reagent (5 mol%), DIPEA 4 equiv and HCO_2_H 4 equiv in CHCl_3_ and using Green LED lights. Under these conditions we proceed to study the scope of the reaction, using several tetrahydroisoquinolines (Scheme 3).

**SCHEME 4:**
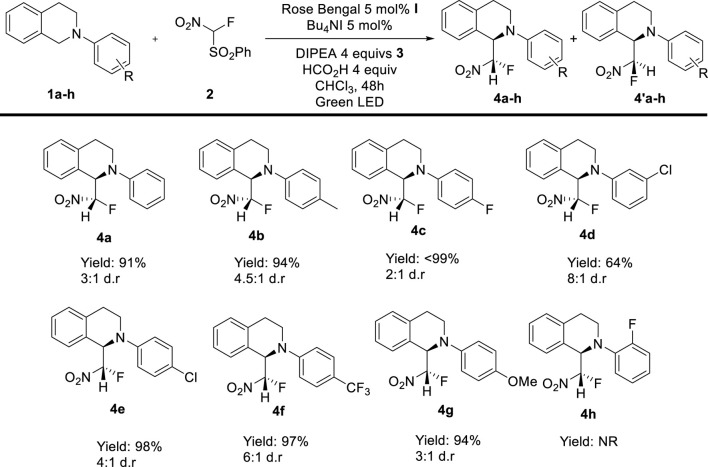
Scope of the reaction.

As it is shown, the reaction generally works with good yields and moderate to good diastereoselectivities. Remarkably, the reaction only yielded the desulfonylated product. Tetrahydroisoquinolines with different aromatic substituents in the Nitrogen were tested. The reaction was compatible with several functional groups like *p*-Me (**4b**, 94% 4.5:1 d.r.), halogens (*p*-F (**4c**, 99% 2:1 d.r.), *m*-Cl (**4d**, 64% 8:1 d.r.), *p*-Cl (**4e**, 98% 4:1 d.r.)), electrowithdrawing groups: p-CF_3_ (**4f**, 94% 6:1 d.r.), and electrodonating grous *p*-OMe (**4g**, 94% 3:1 d.r.) and the only limitation seems to be ortho substituents as the reaction of *o*-F compound **4h** did not render any product (Scheme 4).

Remarkably the relative configuration of the major diastereomer was determined by X-Ray analysis of a single crystal of **4c** and **4f**.

### 3.3 Mechanism discussion

To delineate the reaction pathway, control experiments were performed. Adding the fluoronitro (phenylsulfonyl)methane (FNSM) reagent under reaction conditions without tetrahydroisoquinoline yielded no desulfonylated product **12**, suggesting desulfonylation does not precede the addition. Conversely, subjecting the sulfonylated intermediate **13** to the standard reaction conditions yielded the final product, clearly indicating that desulfonylation occurs *after* the addition step.

Once we determined the reaction pathway, we check the need of the addition of DIPEA. When the reaction is conducted in the absence of DIPEA, only **13** is obtained. Remarkably the addition of the DIPEA (**3**) to the reaction mixture with sulfonylated product lead to the desulfonylation product **4f**, establishing DIPEA (**3**) as a reductant.

**SCHEME 5:**
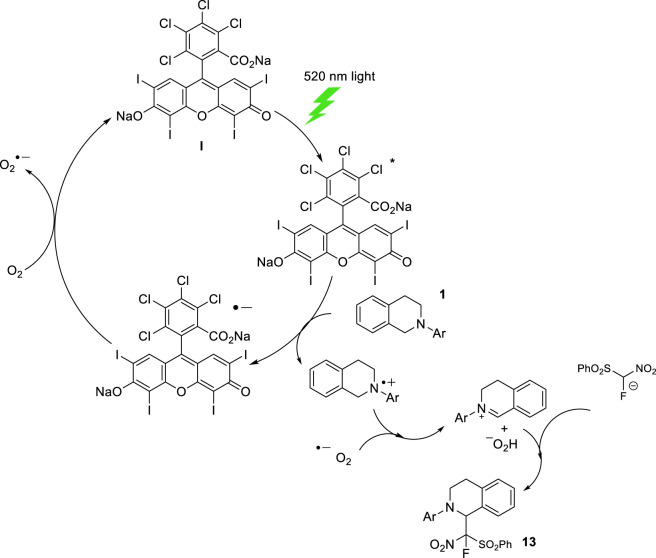
Proposed Mechanism for the addition of FNSM to tetrahydroisoquinolines.

Based on this evidence, we propose a mechanism involving two catalytic cycles. In the first cycle, photoexcited Rose Bengal undergoes oxidative quenching by the tetrahydroisoquinoline **1** via a single electron transfer (SET) process. The resulting radical cation reacts with the superoxide radical anion (generated by reoxidation of the dye) to form the iminium. Concurrently, deprotonated FNSM reacts with iminium to form the sulfonylated intermediate **13** (Scheme 5).

**SCHEME 6:**
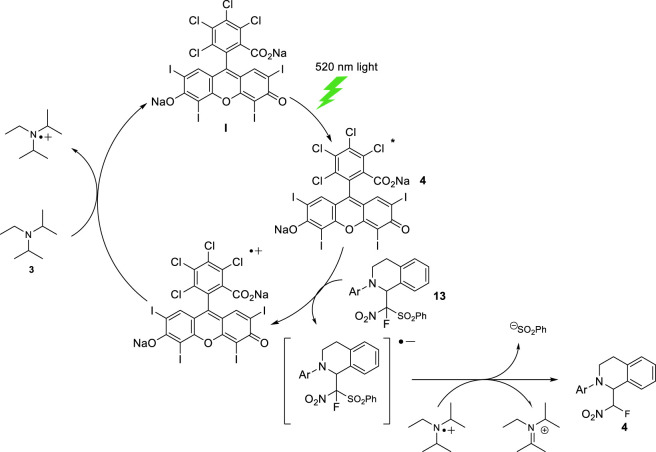
Proposed mechanism for the sulfonylation reaction.

In the second catalytic cycle, the Rose Bengal **I**, accepts a photon from visible light source (Green LEDs) and is promoted to an excited state. Next, the sulfonylated product **13** can oxidatively quench the excited state of the Rose Bengal **I**, leading to the anion radical of **13**. The radical anion dye is reduced to the ground state dye by Diisopropylethylamine **3** and the resulting cation radical of diisopropylethylamine reacts with the radical anion of **13** to get the DIPEA in his iminium form and the final desulfonylated product **3**. This mechanism is in agreement with that previously reported by (Scheme 6).

## 4 Conclusion

In summary, we have successfully developed a novel and sustainable organocatalytic methodology for the formal addition of fluoronitromethane to tetrahydroisoquinolines via a Cross-Dehydrogenative Coupling (CDC) process. By utilizing Rose Bengal as a cost-effective, metal-free photocatalyst under Green LED irradiation and aerobic conditions, we achieved the synthesis of valuable fluorinated scaffolds in good yields and with moderate to good diastereoselectivities. The protocol demonstrated broad functional group tolerance, compatible with electron-withdrawing and electron-donating substituents, although ortho-substitution was found to inhibit the transformation.

Detailed mechanistic investigations, including control experiments, elucidated a unique cascade pathway. We confirmed that the reaction does not proceed via the direct addition of fluoronitromethane; rather, it involves the initial CDC addition of $\alpha$-fluoro-$\alpha$-nitro (phenylsulfonyl)methane followed by an *in situ* photocatalytic desulfonylation. The dual role of the organic dye was highlighted, operating in two distinct catalytic cycles to facilitate both the oxidative generation of the iminium intermediate and the subsequent reductive desulfonylation, for which DIPEA serves as a crucial reductant. This methodology offers a mild, atom-economical, and environmentally friendly alternative for the late-stage introduction of fluorine into *N*-heterocycles, addressing current challenges in sustainable synthesis.

## Data Availability

The original contributions presented in the study are included in the article/supplementary material, further inquiries can be directed to the corresponding author.
